# Supplementary figures and images for: Linkage to HIV care and hypertension and diabetes control in rural South Africa: Results from the population-based Vukuzazi Study
Source: PLOS Glob Public Health. 2022 Nov 2;2(11):e0001221. doi: 10.1371/journal.pgph.0001221 (PMC10021540; doi:10.1371/journal.pgph.0001221)

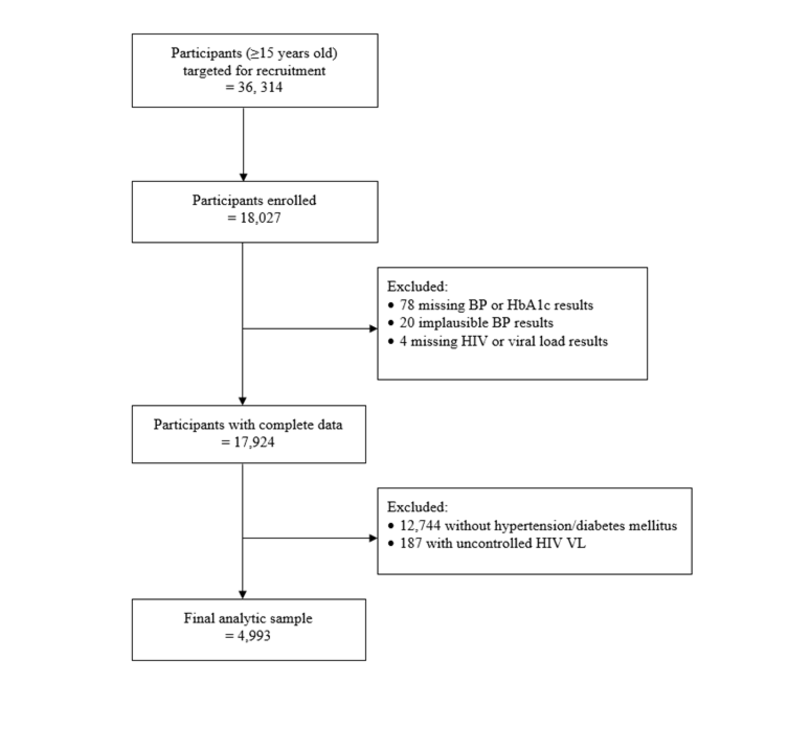

Supplement: S1 Fig — (TIF) [file pgph.0001221.s001.tif]

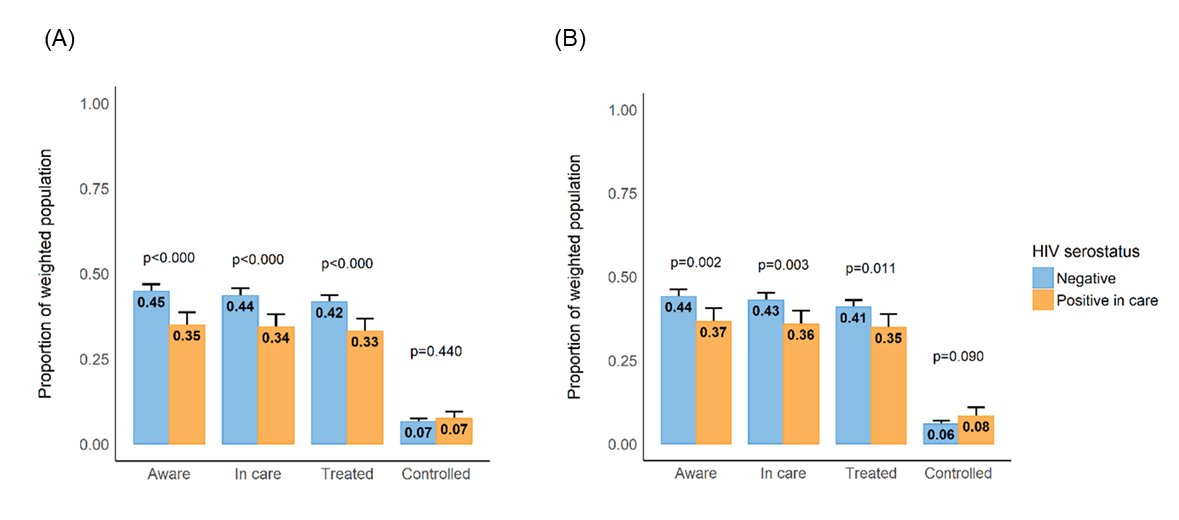

Supplement: S2 Fig — a. Minimally adjusted diabetes care cascade. * Estimates adjusted for age and sex only and include inverse probability of sampling weights. b. Fully adjusted diabetes care cascade. *Estimates adjusted for age, sex, BMI, education, smoking status and wealth tertile, and include inverse probability of sampling weights. (TIF) [file pgph.0001221.s002.tif]

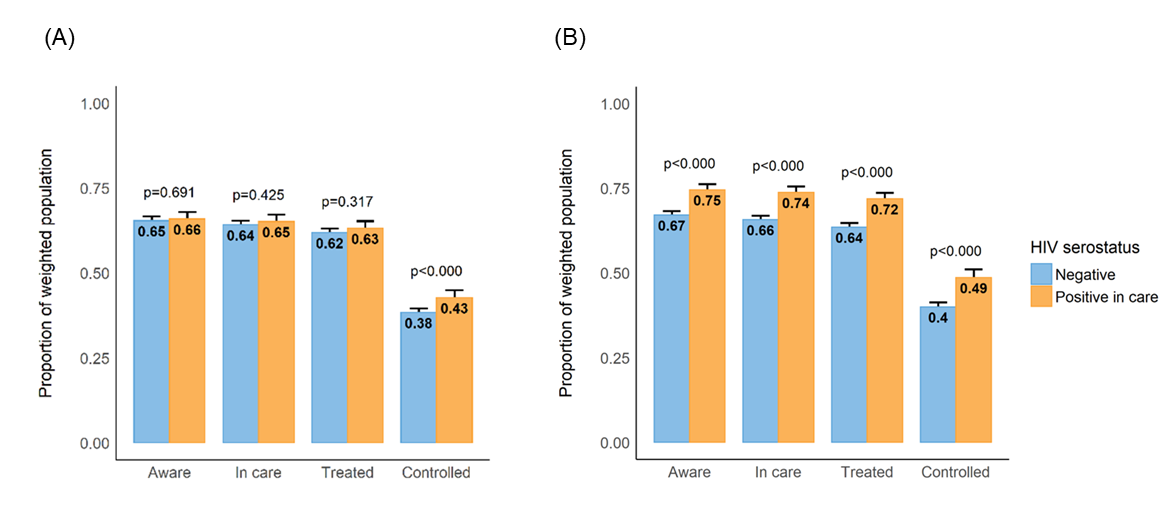

Supplement: S3 Fig — a. Minimally adjusted hypertension care cascade. *Estimates adjusted for age and sex only and include inverse probability of sampling weights. b. Fully adjusted hypertension care cascade. * Estimates adjusted for age, sex, BMI, wealth tertile, education and smoking status and include inverse probability of sampling weights. (TIF) [file pgph.0001221.s003.tif]

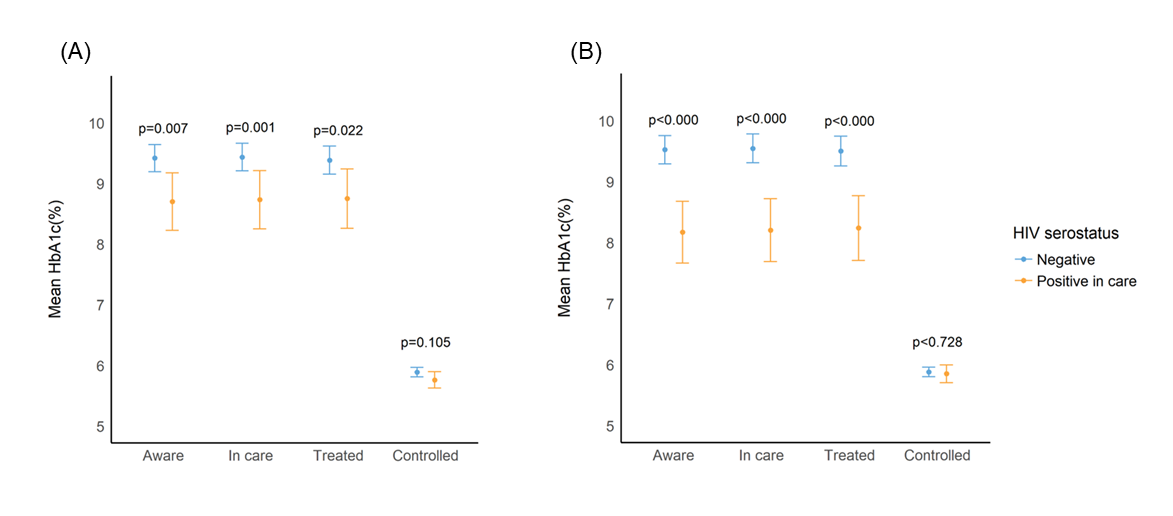

Supplement: S4 Fig — a. Partially adjusted predicted mean HbA1c. * Estimates adjusted for age and sex only and include inverse probability of sampling weights. S4b. Fully adjusted predicted mean HbA1c. * Estimates adjusted for age, sex, BMI, education, smoking status and wealth tertile, and include inverse probability of sampling weights. (TIF) [file pgph.0001221.s004.tif]

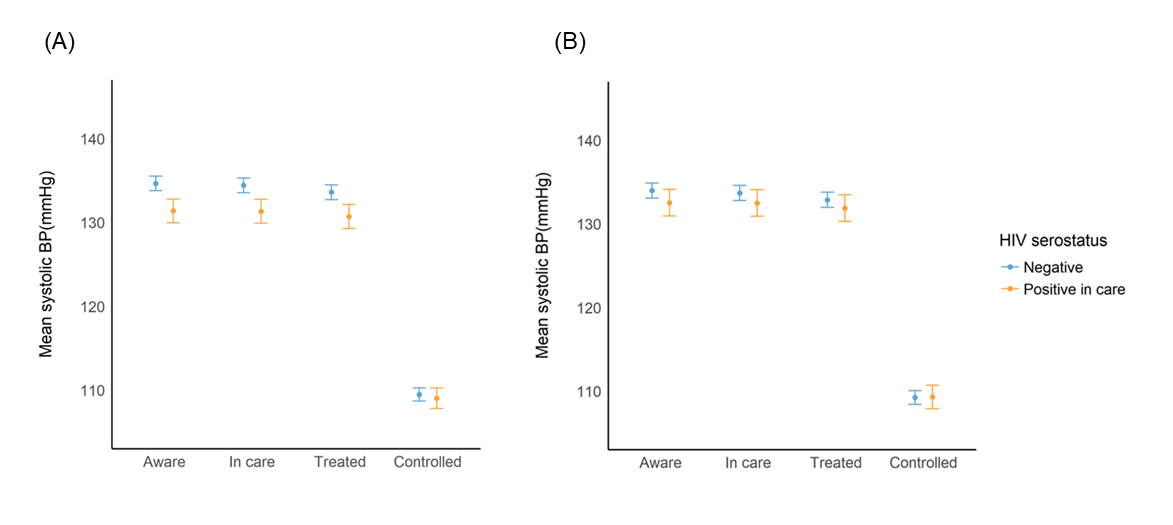

Supplement: S5 Fig — a. Minimally adjusted predicted mean SBP. * Estimates adjusted for age and sex only and include inverse probability of sampling weight. b. Fully adjusted predicted mean SBP. * Estimates adjusted for age, sex, BMI, education, smoking status and wealth tertile, and include inverse probability of sampling weights. (TIF) [file pgph.0001221.s005.tif]
