# Supplementary material for: Linkage to HIV care and hypertension and diabetes control in rural South Africa: Results from the population-based Vukuzazi Study
Source: PLOS Glob Public Health. 2022 Nov 2;2(11):e0001221. doi: 10.1371/journal.pgph.0001221 (PMC10021540; doi:10.1371/journal.pgph.0001221)
Supplement: S1 Table — a Individual participant characteristics used to estimate sampling weights were age, sex, marital status, educational attainment, household asset ownership, distance from Vukuzazi study site, self-reported health status, alcohol use, self-reported HIV infection status and employment. b For participants aged ≥18 years old. (DOCX) [file pgph.0001221.s006.docx]

| **Characteristic** | **Unweighted Sample** | **Weighted**  **Sample^a^** | **True Population Estimate** |
| --- | --- | --- | --- |
|  | (*n = 18,027* ) | (*n = 36,293*) | (*n = 36,314*) |
| Male | 32% | 41% | 42% |
| Female | 68% | 59% | 58% |
| Mean age (years) | 40.4 | 37.4 | 37.5 |
| Age category (years) |  |  |  |
| <25 | 28% | 31% | 31% |
| 25 - 44 | 33% | 37% | 38% |
| 45 – 64 | 25% | 22% | 22% |
| ≥65 | 14% | 10% | 9% |
| Marital Status |  |  |  |
| Single (never married) | 69% | 74% | 74% |
| Married/Informal union | 17% | 15% | 15% |
| Widowed/divorced/separated | 14% | 11% | 11% |
| Highest Attained Formal Education |  |  |  |
| Primary or less | 34% | 30% | 27% |
| Secondary | 61% | 66% | 66% |
| Post-secondary | 4.2% | 4.4% | 6.9% |
| Employment Status |  |  |  |
| Unemployed | 77% | 75% | 67% |
| Employed part-time | 4.1% | 4.4% | 4.5% |
| Employed full-time | 19% | 20% | 28% |
| Household Wealth Tertiles |  |  |  |
| Low | 37% | 37% | 33% |
| Middle | 35% | 35% | 33% |
| High | 28% | 28% | 33% |
