# Supplementary material for: Linkage to HIV care and hypertension and diabetes control in rural South Africa: Results from the population-based Vukuzazi Study
Source: PLOS Glob Public Health. 2022 Nov 2;2(11):e0001221. doi: 10.1371/journal.pgph.0001221 (PMC10021540; doi:10.1371/journal.pgph.0001221)
Supplement: S2 Table — a Values presented as means (95% CI) or number (percent). b For participants aged ≥18 years old. (DOCX) [file pgph.0001221.s007.docx]

| **Characteristic^a^** | **HIV Negative**  **(*n = 3,798*)** | **HIV on ART**  **(*n = 1,290*)** | **P value** | **Overall**  **(*n = 5,088*)** |
| --- | --- | --- | --- | --- |
| Proportion (%) |  |  |  |  |
| Female | 2,899 (76%) | 1,032 (80%) | 0.007 | 3,931 (77%) |
| Marital Status |  |  |  |  |
| Single (never married) | 845 (35%) | 506 (67%) | <0.001 | 1,351 (43%) |
| Married/Informal union | 791 (33%) | 113 (15%) |  | 904 (28%) |
| Widowed/divorced/separated | 782 (32%) | 140 (18%) |  | 922 (29%) |
| Mean Age *(years)* | 62.0 (53.0 - 71.0) | 52.0 (42.0 - 59.0) | <0.001 | 59.0 (49.0 - 69.0) |
| <25 | 172 (4.5%) | 22 (1.7%) | <0.001 | 194 (3.8%) |
| 25 - 44 | 374 (9.8%) | 363 (28%) |  | 737 (14%) |
| 45 – 64 | 1,607 (42%) | 750 (58%) |  | 2,357 (46%) |
| ≥65 | 1,645 (43%) | 155 (12%) |  | 1,800 (35%) |
| Highest Attained Formal Education |  |  |  |  |
| Primary or less | 2,445 (68%) | 579 (48%) | <0.001 | 3,024 (63%) |
| Secondary | 1,046 (29%) | 567 (47%) |  | 1,613 (34%) |
| Post-secondary | 101 (2.8%) | 70 (5.8%) |  | 171 (3.6%) |
| Household Wealth Tertiles |  |  |  |  |
| Low | 1,246 (34%) | 455 (36%) | 0.074 | 1,701 (34%) |
| Middle | 1,262 (34%) | 428 (34%) |  | 1,690 (34%) |
| High | 1,202 (32%) | 365 (29%) |  | 1,567 (32%) |
| Employment Status^b^ |  |  |  |  |
| Unemployed | 3,076 (84%) | 926 (74%) | <0.001 | 4,002 (82%) |
| Employed part-time | 112 (3.1%) | 56 (4.4%) |  | 168 (3.4%) |
| Employed full-time | 453 (12%) | 277 (22%) |  | 730 (15%) |
