# Supplementary material for: Linkage to HIV care and hypertension and diabetes control in rural South Africa: Results from the population-based Vukuzazi Study
Source: PLOS Glob Public Health. 2022 Nov 2;2(11):e0001221. doi: 10.1371/journal.pgph.0001221 (PMC10021540; doi:10.1371/journal.pgph.0001221)
Supplement: S3 Table — a Values presented as means (95% CI) or number (%) or bmedian (interquartile range). c Previous CVD (cardiovascular disease) = self-reported previous diagnosis of heart failure, stroke, myocardial infarction. (DOCX) [file pgph.0001221.s008.docx]

| **Characteristic^a^** | **HIV Negative**  **(*n = 3,798*)** | **HIV on ART**  **(*n = 1,290*)** | **P value** | **Overall**  **(*n = 5,088*)** |
| --- | --- | --- | --- | --- |
| **Obesity** |  |  |  |  |
| Mean BMI *(kg/m^2^)* | 31.2 (26.0 - 36.9) | 30.0 (25.0 - 35.6) | <0.001 | 30.8 (25.7 - 36.5) |
| Underweight | 74 (2.0%) | 34 (2.7%) |  | 108 (2.2%) |
| Normal | 686 (19%) | 287 (22%) |  | 973 (20%) |
| Overweight | 873 (24%) | 314 (25%) |  | 1,187 (24%) |
| Obese | 2,050 (56%) | 641 (50%) | 0.002 | 2,691 (54%) |
| Mean Waist Circumference *(cm)* | 98.0 (86.0 - 109.0) | 95.0 (84.0 - 108.0) | <0.001 | 97.0 (86.0 - 109.0) |
| Increased | 2,461 (65%) | 800 (62%) | 0.045 | 3,261 (64%) |
| **Diabetes Mellitus** |  |  |  |  |
| Mean HbA1c *(%)* | 6.0 (5.7 - 6.7) | 5.9 (5.6 - 6.5) | <0.001 | 6.0 (5.6 - 6.6) |
| Normal (<5.7%) | 901 (24%) | 400 (31%) |  | 1,301 (26%) |
| Pre-diabetic (5.7 – 6.4%) | 1,633 (43%) | 564 (44%) |  | 2,197 (43%) |
| Raised (≥6.5%) | 1,263 (33%) | 326 (25%) | <0.001 | 1,589 (31%) |
| Current diabetes mellitus | 1,352 (36%) | 354 (27%) | <0.001 | 1,706 (34%) |
| **Hypertension** |  |  |  |  |
| Mean Systolic BP *(mmHg)* | 134.5 (122.0 - 148.5) | 130.0 (119.0 - 143.0) | <0.001 | 133.5 (121.0 - 147.0) |
| Mean Diastolic BP *(mmHg)* | 81.5 (73.5 - 91.0) | 82.8 (75.0 - 91.5) | 0.008 | 82.0 (73.5 - 91.0) |
| Normal | 647 (17%) | 249 (19%) |  | 896 (18%) |
| Pre-hypertension | 1,156 (30%) | 421 (33%) |  | 1,577 (31%) |
| Stage 1 hypertension | 1,360 (36%) | 466 (36%) |  | 1,826 (36%) |
| Stage 2 hypertension | 633 (17%) | 154 (12%) | <0.001 | 787 (15%) |
| Current hypertension | 3,382 (89%) | 1,129 (88%) | 0.13 | 4,511 (89%) |
| **Smoking** |  |  |  |  |
| Never | 3,586 (94%) | 1,214 (94%) |  | 4,800 (94%) |
| Former | 43 (1.1%) | 12 (0.9%) |  | 55 (1.1%) |
| Current | 169 (4.4%) | 64 (5.0%) |  | 233 (4.6%) |
| Previous CVD^c^ | 229 (6.0%) | 86 (6.7%) | 0.400 | 315 (6.2%) |
| **Comorbidity (hypertension and diabetes)** |  |  |  |  |
| Comorbidity | 936 (25%) | 193 (15%) | <0.001 | 1,129 (22%) |
| **HIV Disease** |  |  |  |  |
| Current CD4+ count *(cells/mL)^b^* | - | 748.0 (541.8 - 967.2) | - | 750.0 (542.0 - 973.0) |
