# Supplementary material for: Linkage to HIV care and hypertension and diabetes control in rural South Africa: Results from the population-based Vukuzazi Study
Source: PLOS Glob Public Health. 2022 Nov 2;2(11):e0001221. doi: 10.1371/journal.pgph.0001221 (PMC10021540; doi:10.1371/journal.pgph.0001221)
Supplement: S4 Table — a Values presented as means (95% CI) or number (%) or bmedian (interquartile range). c Previous CVD (cardiovascular disease) = self-reported previous diagnosis of heart failure, stroke, or myocardial infarction. (DOCX) [file pgph.0001221.s009.docx]

| **Characteristic^a^** | **HIV Positive** | | **P value** | **Overall**  **(n = 1,286** |
| --- | --- | --- | --- | --- |
|  | **Successful ART**  **(n = 1,195)** | **Failing ART**  **(n = 91)** |  |  |
| **Obesity** | | | | |
| Mean BMI *(kg/m^2^)* | 30.1 (25.1 - 35.6) | 28.1 (23.4 - 36.4) | 0.200 | 30.0 (25.0 - 35.6) |
| Underweight | 31 (2.6%) | 3 (3.3%) | 0.200 | 34 (2.7%) |
| Normal | 257 (22%) | 27 (30%) |  | 284 (22%) |
| Overweight | 291 (25%) | 23 (25%) |  | 314 (25%) |
| Obese | 602 (51%) | 38 (42%) |  | 640 (50%) |
| Mean Waist Circumference *(cm)* | 95.0 (85.0 - 108.0) | 92.0 (81.5 - 103.5) | 0.140 | 95.0 (84.0 - 108.0) |
| Increased | 898 (75%) | 59 (65%) | 0.028 | 957 (75%) |
| **Diabetes Mellitus** | | | | |
| Mean HbA1c *(%)* | 5.9 (5.6 -6.4) | 6.0 (5.5 - 6.6) | 0.600 | 5.9 (5.5 - 6.5) |
| Normal (<5.7%) | 368 (31%) | 31 (34%) | 0.300 | 399 (31%) |
| Pre-diabetic (5.7 – 6.4%) | 529 (44%) | 33 (36%) |  | 562 (44%) |
| Raised (≥6.5%) | 298 (25%) | 27 (30%) |  | 325 (25%) |
| Current diabetes mellitus | 326 (27%) | 27 (30%) | 0.600 | 353 (27%) |
| **Hypertension** | | | | |
| Mean Systolic BP *(mmHg)* | 130.0 (119.0 - 143.0) | 131.0 (121.2 - 142.0) | 0.900 | 130.0 (119.0 - 143.0) |
| Mean Diastolic BP *(mmHg)* | 82.5 (74.5 - 91.5) | 85.8 (78.6 - 92.4) | 0.073 | 83.0 (75.0 -91.5) |
| Normal | 232 (19%) | 15 (16%) | 0.017 | 247 (19%) |
| Pre-hypertension | 395 (33%) | 25 (27%) |  | 420 (33%) |
| Stage 1 hypertension | 419 (35%) | 46 (51%) |  | 465 (36%) |
| Stage 2 hypertension | 149 (12%) | 5 (5.5%) |  | 154 (12%) |
| Current hypertension | 1,052 (88%) | 74 (81%) | 0.061 | 1,126 (88%) |
| **Smoking** | | | | |
| Never | 1,128 (94%) | 82 (90%) | 0.200 | 1,210 (94%) |
| Former | 11 (0.9%) | 1 (1.1%) |  | 12 (0.9%) |
| Current | 56 (4.7%) | 8 (8.8%) |  | 64 (5.0%) |
| Previous CVD^c^ | 77 (6.4%) | 9 (9.9%) | 0.200 | 86 (6.7%) |
| **Comorbidity (hypertension AND diabetes)** | | | | |
| Comorbidity | 183 (15%) | 10 (11%) | 0.300 | 193 (15%) |
| **HIV Disease** | | | | |
| Current CD4+ count *(cells/mL)* ^b^ | 763.0 (562.0 - 979.0) | 456.0 (283.0 - 717.5) | <0.001 | 748.5 (542.0 - 968.2) |
| Current viral *(copies/mL)*^b^ | ≤40 | 1,045.0 (134.5 - 12,419.0) | <0.001 | 2.0 (2.0 - 2.0) |
