# Supplementary material for: Linkage to HIV care and hypertension and diabetes control in rural South Africa: Results from the population-based Vukuzazi Study
Source: PLOS Glob Public Health. 2022 Nov 2;2(11):e0001221. doi: 10.1371/journal.pgph.0001221 (PMC10021540; doi:10.1371/journal.pgph.0001221)
Supplement: Attachment — Submitted filename: Response to reviewers_2Oct2022.docx [file pgph.0001221.s010.docx]

1518 Clifton Road, Atlanta, Georgia 30322

October 3, 2022

Academic Editor

PLoS Global Health

Dear Dr. Anil Gumber,

**RE: PGPH-D-22-01159. Linkage to HIV care and hypertension and diabetes control in rural South Africa: results from the population-based Vukuzazi Study.**

Our above referenced manuscript refers. Thanks to the reviewers for their time and invaluable feedback they have provided to our submission. Please see below our responses to their comments.

**Reviewer #1**

1. *Research topic: the research topic needs to be reframed. It’s not catchy and leaves room for varied interpretation and meanings for readers*. *This statement ....'collecting blood pressure' should be written as checking blood pressure*. *It will be nice if the size of the sample population is stated in the method section of the abstract*.

These are invaluable comments. However, we believe they are concerned with style, which will necessarily vary with personal preference.

2. *Line 90. Please quote figures and cite reference to back your claim....'in one of the world's high HIV burden setting'.*

New references (number 33 and 34) have been added.

3. *I guess there are real or perceived benefits of the HIV care programs for NCD care to the patients, health institutions and to governments. Please elaborate on this using current literature.*

The paragraph between lines 75-84 and its attendant references is in fact an elaboration of these supposed benefits. The same paragraph also cites the example of South African government policy on this matter.

4. *To help readers appreciate the strength of your claim please state the hypothesis for the study*.

This is explicitly stated in lines 91-94: “We hypothesized that PWH with a suppressed viral load would have evidence of improved indicators along the NCD cascade of care, supporting a role of expanding the HIV care model for management of chronic diseases more broadly.”

5. *The study design is not well stated. Provide a brief description of the sample population, the inclusion and exclusion criteria is not well described. For readers who do not know uMkhanyakude district or rural KwaZulu Natal, what made this district the most appropriate for this study. How many sub-districts, towns and households were involved in this study.*

These details are comprehensively described under the heading **Study design, population, and setting (**lines 97-108). We believe the depth of detail that we have provided will allow our readers to interpret our results without prejudice. We have also included references for readers who might require additional information about uMkhanyakude district, KwaZulu Natal.

6. *Of the 36,314 residents that were invited for the study, how many satisfied the inclusion criteria and were involved in the study. this information needs to be captured under this sub-heading*.

This information is portrayed in the flow chart (**Supplementary Figure 1**) and amply explained under the section **Derivation of analytic sample.**

7. *Sampling: The manuscript does not provide details of the sampling procedure used including sampling method, sampling technique and recruitment procedures. Based on the information provided, it seems the authors used convenience sample making this study susceptible to selection bias. specifically, what sampling methods were used to select the sub-districts, towns, households and sample population.*

We do indicate that our study was a population-wide survey covering all households in the catchment or study area. This is explained under the section **Study design, population, and setting (**lines 97-108). We also describe under the section **Data analysis** our use of inverse probability sampling weights to account for selection (or participation) bias.

8. *How were the participants recruited, were they recruited to enroll in the integrated HIV and NCD clinical care or participants who were already enrolled in the integrated HIV and NCD care were recruited??? please be clear on this*.

Participants in the Vukuzazi study were recruited from their households as is explained in the section **Study design, population, and setting (**lines 97-108)).

9. *Ethics: Since the nature of your study involves invasive procedures and human subjects, Kindly described how you adhered to the ethical principles of the Declaration of Helsinki. Specifically, how you addressed; Anonymity, privacy, confidentiality, protection from harm, risk and injury, beneficence, voluntary participation, Disclosure of identified ill-health or unexpected test results or outcome. Was the services of a clinical psychologist sought for persons that might suffer psychological stress or pain following disclosure?*

Because of word limitations on manuscripts, we have necessarily omitted some details, specifically those pertaining to descriptions of study standard operating procedures (SOPs). Nonetheless, we have added the sentence quoted below to further assure readers that our study was ethically sound.

“All study activities were conducted in keeping with the principles of the Declaration of Helsinki, and had prior approval of the Africa Health Research Institute (AHRI) review board.” Line 108-109.

10. *Data collection: state the day, month and year this study was initiated and for how long it lasted*.

This is stated [May 2018 – March 2020] as under the section **Derivation of analytic sample** in line 168.

11. *Results: General characteristics: could you just report on the percentage and ignore the confidence interval.*

*Diabetes mellitus care cascade: could you elaborate on what 'diff' means. it's difficult to comprehend. Are we dealing with odds ratio. You can check this article for reporting on odds ratio; Szumilas M. Explaining odds ratios. J Can Acad Adolesc Psychiatry;2010 Aug, 19(3): 227-9.*

We have adhered to the STROBE recommendations on the reporting of results. Each table and figure of results in our manuscript explicitly states the effect estimates that are being reported.

12. *Can you elaborate on whether you dichotomized controlled hypertension and glycemic control? Should the readers assume they are categorical or continuous? please state so the method section.* *Fig 2a,2b,4a,4b are very faint. Make it more clearer.*

Readers need not assume anything as all outcomes are explicitly defined in the methods section, under **Data Analysis.** We do not see how these explicit definitions can be further improved upon. The figures are high resolution and we hope they are clearer now.

13. *Discussion: Line 315: Be consistent with your intext-reference style. Part of your justification for this study was the fact that economic justification for HIV/NCD integration in SSA remains unproven. Yet, information on how your study addressed this gap is not captured neither is it extensively discussed in your study*.

The in-text referencing style in question [line 315] is in keeping with the guidelines of the PLoS Global Health journal.

Our reference to the lack of economic justification for HIV/NCDs integration was made in the broader context of a sparse evidence base – epidemiological, economic, clinical, etc., to support this policy recommendation. However, the particular hypothesis and thus the focus of our study was epidemiological and not economical.

14. *Conclusion: Line 352 (now line 362). Please limit your conclusion the rural district of KwaZulu Natal. Line 354, I guess the statement beginning with 'Whereas....... shouldn't have ended the way it did. Please check and revise.*

This is precisely what our conclusion is: “Engagement in HIV care with successful viral suppression was not associated with meaningful improvements in hypertension or diabetes care for PLWH **in a low-income and rural district of South Africa** where both HIV and NCDs are common”. Line 362.

15. *Limitation: well stated but please provide a recommended focus for future studies. General comment: the supplementary material was not a readable file and so could not be reviewed.*

This is precisely what we have done: “…much work is needed to understand the optimal design and implementation of integrating additional chronic disease services into HIV programs in rural South Africa; as well as to extend the lessons learned from the HIV care program to the general population.” Lines 366-368.

Judging from their comments on supplementary material, we assume that the second reviewer was able to access the supplementary file. The failure, if it is such, to access the supplementary file cannot therefore be because the file is problematic.

**Reviewer #2**

Major comments:

**1**. *The HIV+/suppressed group definition was a little unclear. “Linkage to care” (line 131) was defined as enrolled in ART plus virologically suppressed, and then the “primary analysis” (line 136) compared HIV-negative and HIV+/suppressed individuals. I am assuming the HIV+/suppressed comparison group is not solely based on viral load but actually means the group was “linked to care” and suppressed? This raises several follow-up questions:*

**a***. Was enrolment in ongoing ART self-reported or defined through medical records with TIER.net? In either case, is it possible that ART enrolment information could be missing or mis-reported, in which case it may be helpful to consider a sensitivity analysis that ignores ART enrolment entirely and solely defines the HIV+/Suppressed group on viral load result*.

To avoid ambiguity, we now consistently use “HIV positive/suppressed” to refer to HIV infected persons who are enrolled in ART (≥6 months) and are virally suppressed. ART enrolment was defined using TIER.net records.

Our study, like all studies, was/is vulnerable to missing data and misclassification bias. We acknowledge this under the **Limitations** section. Notwithstanding, our interest was in looking at the benefits of linkage to HIV/ART care on NCD outcomes. Thus ignoring ART enrolment, while an interesting comparator group, would not be appropriate for the question at hand. Equally, it would not necessarily solve the ever likely problem of missing and/or misclassified data.

**b**. *I was surprised to see only 187 individuals had unsuppressed HIV (line 171), especially with such a low cut-off of 40 copies/mL. Is this because you already limited the HIV sample to those enrolled in ART? It would be helpful to see or read a clearer breakdown of HIV in the population, perhaps with a table or a chart related to the entire study participation: % HIV+, among those who are positive the % with viral suppression and the % enrolled in ART (including how exactly each of these are defined).*

District-wide data on the HIV care continuum for the study area have been published previously, and so we chose not to include them in the present manuscript. We have, however, since made explicit reference to these published data by including the following, “*…and the HIV care continuum for the district has been reported elsewhere*[35]” in *lines 104-105.*

***c***. *The HIV+/Suppressed comparison group is later written as “HIV+/successful ART” group (for example in Table 2). The inconsistent terminology to refer to this group added to the lack of clarity. Similarly, in line 294, PWH with and without viral suppression is mentioned with reference to Supplementary Table 4, but Supplementary Table 4 compares “successful ART” and “Failing ART” with no mention of those NOT on ART, which would likely be an important virally unsuppressed group of people*.

We have rectified this as explained under point ***1.a*** above.

***d***. *The sensitivity analysis including everyone enrolled in ART regardless of viral load was important and helpful*.

Noted, thank you.

**2**. Inverse probability weights for the participation in the Vukuzazi study:
***a***. *Weighting is briefly described using age and sex, but the participation rate in the study is not stated in the text. It looks like in Supplementary Table 1 that the full population invited to participate in the study was 36,314. Were all of these individuals invited to participate or was fieldwork closed early in March 2020? Given that 18,027 participated in the Vukuzazi study it appears the response rate to the survey was ~50%. It would be helpful to more directly address this as a limitation of the study*.

We have added the following explicit acknowledgement to the **Limitations** section, “*The present analysis was undertaken at nearly 50% enrolment of target eligible sample, potentially threatening the external validity of our results from likely selection bias*.” in lines 356-358.

***b***. *Age and sex were included in the IPW models – why not other characteristics (marital status, education, employment, wealth)? It appears you have this data available of the full population*.

Indeed, our weights could have incorporated the additional characteristics that you highlight. However, we found that the age and sex IPW model performed well in formal testing as summarized in Supplementary Table 1.

***c***. *Depending on the participation rate to the blood sample collection, I would also consider weighting to account for lack of consent to blood collection*.

Response rates to the interviews, anthropometric examination and blood collection were similar because giving blood was required for study participation. This obviated the need for different weights.

3. *Response rate to venous blood collection: The paper says that venous blood HbA1c level was used to define the diabetic population, yet in many population based studies response rate to venous blood collection can be quite low. Can the authors include the response rate to blood collection and comment on how those who give blood may differ from those who did not? Assuming the response rate to blood pressure measurement is much higher than venous blood collection, does this lead to differences in the populations who have diabetes data vs. hypertension data, and could any differences observed here relate to the different findings in the diabetes care cascade vs. the hypertension care cascade? Or was giving blood required for the study participation, which would explain a response rate of ~50%?*

Please see response ***2.c*** above.

4. *Given the large sample size, I would be interested to see if results differ when stratified by age group. Is there any evidence that the NCD care is better in older HIV+ populations?*

This is an important question that was the focus of a previous report from our group [reference 35].

Minor comments:
1. *Conclusion of the study – I agree that overall results indicate poor diabetes and hypertension care and need for primary healthcare strengthening. Yet, does the fact that disease control was better among the HIV+/suppressed group (and therefore in theory associated with more frequent contact with the healthcare system through HIV care) indicate integration of HIV and NCD care could be a promising way to improve NCD care for the HIV-positive population?*

Your observations are spot on. Perhaps it comes down to whether one is more *glass-is-half-full* or is *glass-is-half-empty*. We argue that the observed statistically significant improvements in rates of disease control may not in fact translate into significant clinical or population health advantage for persons living with HIV over the general population. For example, the 3% higher rates of diabetes control for HIV+/suppressed than HIV- occur against the backdrop of overall diabetes control rates of 6.8% in this population [reference 35]. Hence, we argue that ”[Overall], *our findings call into question the successful track record of expanding HIV care platforms to address NCDs in this setting*.” Line 317-318. Perhaps our readers will be, as you are, more charitable in their interpretation than we are.

**2**. *Read through for some awkward wording and typos. For example line 64: “continuity of care over across the lifespan” and line 78-79 “beginning in 2011… in 2011”.*

Thank you. Corrections have been made.

*3*. *Line 161: Suggest deleting “with a 2-sided p value of < 0.05 considered statistically significant.” Confidence intervals are used appropriately throughout the text and ok to include the p-values in tables, but it is not necessary to define a “statistically significant” cut-off*.

Thank you, and noted.

Once again, we thank you for your time to review our work. We hope that the edits that we have effected are at least satisfactory.

Sincerely,

Itai M Magodoro, Post-Doctoral Fellow
